# A Multilevel Physical Activity Intervention Among Chinese Rural Older Adults (Stay Active While Aging): A Study Protocol for a Clustered Randomized Controlled Trial

**DOI:** 10.3389/fpubh.2022.760457

**Published:** 2022-05-03

**Authors:** Nanyan Li, Yufei Wang, Qian Deng, Julinling Hu, Junmin Zhou

**Affiliations:** Department of Health Behavior and Social Medicine, West China School Public Health and West China Fourth Hospital, Sichuan University, Chengdu, China

**Keywords:** rural older adults, physical activity, socio-ecological model, clustered randomized controlled trial, study protocol

## Abstract

**Background:**

Although a large number of studies have confirmed the benefits of physical activity (PA) in preventing age-related diseases and disabilities, a growing number of older people spent more time in sedentary behavior as opposed to PA. To reverse the alarming trend, numerous studies have corroborated the effectiveness of PA interventions in improving PA among older adults. However, such research is scarce in rural China, where a majority of older adults do not meet the PA recommendation. The Stay Active While Aging (SAWA) aimed to conduct an intervention to improve the PA level among older adults in rural China.

**Methods:**

The SAWA is designed as a single-blind, clustered randomized controlled trial carried out in rural Sichuan, China with an 8-week intervention and a 24-month follow-up. The intervention group will receive a multilevel intervention (individual, interpersonal, and community levels), while the control group will not. The primary outcome is the PA level. Secondary outcome measures include sedentary behavior level, self-efficacy, self-regulation, cognitive function, night-time sleep quality, and anthropometry. The difference-in-differences (DID) will be performed to investigate the between-group differences, adjusted for baseline data and covariates.

**Discussion:**

The SAWA trial will provide a multilevel intervention based on the socio-ecologic model among older adults in rural China. We target the PA level and health status changes while also focus on the maintenance of such intervention during 24 months. If the SAWA produces positive results, it will be possible to recommend similar strategies to be implemented in other Chinese older adults and beyond.

**Trial registration:**

ChiCTR2100045653 (https://www.chictr.org.cn/index.aspx).

## Introduction

China houses a large population of older adults, which reached 249 million by the year 2018 (accounting for 17.9% of the total population), and is predicted to increase to 400 million in 2050, making up about 26.9% of the total population (1, 2). Aging is associated with cognitive impairment (3) and a majority of chronic diseases such as cancer, cardiovascular diseases, and mental disorders (4). The surge in the number of older adults and the incidence of age-related diseases (1) will undoubtedly lead to a reduced quality of later life and a heavy financial burden.

A growing number of studies have revealed that physical activity (PA) among older adults is beneficial to healthy aging (5), including reduced incidence of numerous diseases (6) and disability (5). For instance, a systematic review concluded that the incidence of basic activities of daily living disability in physically active older adults was 49% lower than in those with low PA levels (6). Despite the enormous benefits of PA, older people spent more time in sedentary behavior as opposed to PA, and it deteriorated with age (7). According to a systematic review, only 25.30–47.44% of Chinese older adults met the PA recommendation (at least 30 min/day for 5 or more days per week, or an equivalent) (8). In addition, the PA level in those aged 60–79 years (13.38 MET-h/day) was almost half lower than that in those aged 30–59 years (24.35 MET-h/day) in the country (9).

In rural China, the situation is more severe. Rural older adults are less likely to engage in regular PA than their urban counterparts, despite the more drastic aging trend in rural areas (10, 11). A study in Guangdong province of China reported that the rural older adults showed a substantially lower level of PA (3.1 METs), compared with the urban older adults (10.6 METs) (12). Findings from Globe Ageing and Adult Health conducted in China disclosed that average exercise time was 90 min per week among rural older adults, while this number was 210 min among urban older adults (13). It highlights the urgency to improve PA among Chinese rural older adults.

Previous PA interventions demonstrated their effectiveness in improving PA in older adults. Many of them were conducted in developed countries in recent years. A study from the Netherlands revealed that the PA intervention was effective in increasing the days and minutes of PA (14). After an 8-month intervention in America, PA was significantly improved among participants comparing to baseline (15). Nevertheless, current evidence on PA interventions among Chinese older adults is scarce, especially in rural older adults. Therefore, the purpose of the study is to conduct an intervention to improve PA among older adults in rural China.

## Methods

### Design and Setting

The study protocol describes a clustered randomized controlled trial which will be implemented in the rural areas of Sichuan, China. The single-blind trial has been registered on the Chinese Clinical Trial Registry (ChiCTR2100045653).

### Randomization and Sample Size

Multistage random sampling was used, and the random number was generated by the computer program (www.random.org). First, Chengdu was randomly selected from 18 cities in Sichuan Province. Second, Jianyang was selected out of 20 regions in Chengdu. Third, eight villages were randomly selected (Guilin, Jianzheng, Qianfeng, Yixue, Xinsheng, Yijia, Huanglian, Tiane), and these villages were further randomly assigned to either the intervention group or the control group (four villages for each group). There is a separation distance (minimum 4 km) between each of these villages to minimize the risk of contamination.

We hypothesize the effect size of the PA level of the SAWA to be 0.42, based on the prior meta-analysis (16). The calculated sample size for each group is 90 to detect the effect size with a power of 80%, an α of 0.05. Because the randomization occurs at the village level, we need to consider the clustering effect (17).

The sample size (SS) for a clustered RCT is defined by Equation (1):


(1) $$\begin{array}{r} SS\;cluster\;RCT=SS\;standard\;RCT\times DE \end{array}$$

The design effect (DE) is obtained from Equation (2):


(2) $$\begin{array}{r} DE=1+\left(n-1\right)\times ICC \end{array}$$

(3) $$\begin{array}{r} Where:ICC=\frac{\sigma_{between}^{2}}{\sigma_{between}^{2}+\sigma_{within}^{2}\;} \end{array}$$

and *n* = cluster size (number of participants per cluster).

In Equation (3), $\sigma_{between}^{2}$ is the between-cluster variance for the outcome measure and $\sigma_{within}^{2}$ is the within-cluster variance for the outcome measure.

We conducted a pilot study prior to the SAWA to calculate the DE. With a DE of 2.1, we will need a sample size of 189 (90 × 2.1) for each group after factoring in the cluster effects. Taking participation attrition into account, we will screen 20% more participants. Each group will have 227 participants, so the total sample size will be 454 participants for the two groups (intervention group and control group).

### Eligible Criteria

#### Inclusion Criteria

(1) Be 60 years of age or older and
(2) Be able to answer phone calls and
(3) Be able to walk 400 m in 15 min and
(4) Be able to walk without the help of others or crutches and
(5) Be able to complete the Timed Up & Go test (18).

#### Exclusion Criteria

(1) Have a history of stroke, arthritis, Parkinson's disease, severe pneumonia or severe heart disease or
(2) Have severe cognitive or hearing impairment or
(3) Had major surgery in the past 3 years or
(4) Poor control of hypertension or diabetes or
(5) Be receiving cancer treatment or
(6) Have fallen in the past year.

### Interventions

In light of current evidence on PA intervention and follow-up periods (19–21), our project will consist of an 8-week intervention and a 24-month follow-up.

**Figure 1:**
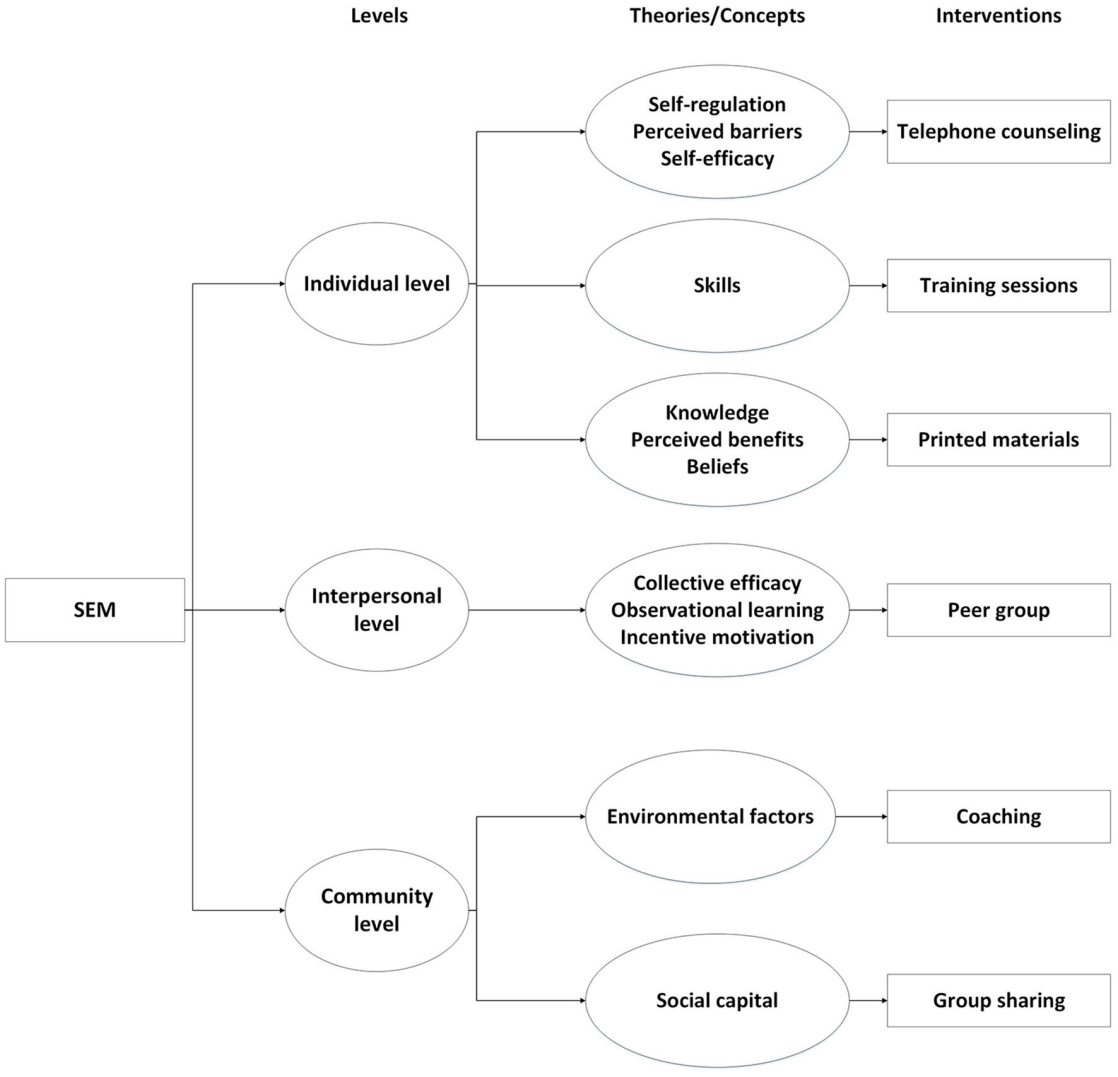
Intervention strategies based on SEM.

During the past two decades, there has been an increasing interest in the socio-ecological model (SEM) (22). The SEM consists of five dimensions: individual, interpersonal, community, organizational, and public policy levels, which are thought to be the determinants of health-related behavior (23, 24). Systematic reviews from intervention studies based on the SEM have revealed the effectiveness of such interventions, indicating that not only PA level was increased, but sedentary time was reduced (25, 26). It is noteworthy that many current studies have condensed the SEM into three levels: individual, interpersonal, and community levels (27, 28). Our detailed interventions based on the three levels (individual, interpersonal, and community levels) are as follows (Figure 1):

#### Individual Level

Individual factors, such as knowledge, beliefs, perceived barriers and benefits, self-regulation, self-efficacy, and skills will be operationalized by telephone counseling, printed materials, and training sessions. Specifically, changes in perceived barriers, self-regulation, and self-efficacy will be achieved by telephone counseling; all the information printed in the materials will lead to improvements in knowledge, perceived benefits, and beliefs towards PA. The skills of PA will be improved through training sessions.

##### Telephone Counseling

During the 8-week intervention period, the participants will receive telephone calls once a week. The telephone call will be used to encourage participants to exercise and provide individualized assistance as follows: (1) set the exercise goals based on their preference and the intensity of their daily exercise, (2) participants will be asked “did you achieve the exercise goals last week?”, (3) investigators will praise the respondents who have achieved the goals, and adjust it according to the global recommendations (29) on PA for older adults, (4) participants who did not achieve the goals will be encouraged, and investigators will assist them to solve the difficulties.

##### Printed Materials

Each participant in the intervention group will receive a booklet, including the potential risks of PA, the benefits of PA, the PA recommendation, local exercise resources for PA, the feasible PA for older adults, and safety tips for performing PA.

##### Training Sessions

The training sessions will be taught by sports experts who are certified to engage in physical education. Stretching exercises and Tai Chi will be mainly included in the courses. There is strong scientific evidence of the benefits of Tai Chi for improving cognitive function and preventing clinical diseases, such as Parkinson's disease, osteoarthritis, and so on (30).

#### Interpersonal Level

With respect to interpersonal level, collective efficacy, observational learning, and incentive motivation will be operationalized through forming peer groups. Collective efficacy will be enhanced by shared group goals and communication. Each participant will learn new information and behaviors from other teammates by observing the behaviors, which will receive positive reinforcement. Further, the well-performing teammates and responsible leaders (encouraging teammates to participate in the group activities) will be rewarded to increase their motivation to participate in PA.

##### Peer group

Peer groups will be formed based on the wishes of participants, with 3–10 participants in each group. Investigators will assist groups to set the group goals based on their common preferences. While organizing group activities, the group leader nominated by the group will also remind the teammates to participate in PA, through which they can communicate and learn from each other. The peer groups will receive telephone calls once a week in the intervention period as well, and will be reminded to achieve the group goals by telephone. The well-performing teammates and responsible leaders will be rewarded.

#### Community Level

At the community level, social capital will be improved through group sharing and coaching. Both of them will be conducted three times on-site at baseline, 4 weeks after baseline, and at the end of the intervention. Participants will be encouraged to join the group sharing led by investigators, through which they can share their experiences and help each other in order to solve problems. Environmental factors will be operationalized through the identification of harmful and beneficial factors in the environment and utilization of environmental resources with the help of coaches.

##### Group Sharing

Both the participants and investigators will join the group sharing. Investigators will lead them to discuss topics related to PA. The purpose of group sharing is to provide participants with opportunities to share experiences of exercise with others, promote mutual trust, and increase social capital. For example, the participant who achieves the goals weekly will be asked “how did you keep exercising when the weather was terrible or the farming was busy?”

##### Coaching

The participants will be guided by coaches to identify barriers to PA in rural settings and factors in the environment that are conducive to exercise. Coaches will also provide guidance on how to comprehensively utilize the environmental resources (e.g., walking paths, open spaces for PA) based on identified facilitating and constraining factors.

### Outcomes

All the outcomes will be collected at baseline, 4 weeks after baseline, end-point, and at 6-, 12- and 24-month follow-up.

#### Primary Outcome

The PA level of participants is the primary research outcome which will be measured by the Physical Activity Scale for the Elderly (PASE), a widely used instrument for older adults. Previous studies demonstrated the validity and reliability of the scale for assessing the PA level of older adults in the Chinese population (31, 32). PA is categorized into three domains in the scale: leisure time PA, household PA, work-related PA. The score of each domain of the PA is calculated by multiplying the weight and frequency (33, 34).

#### Secondary Outcomes

##### Sedentary Behavior

Self-reported sedentary behavior will be assessed through the question, “how much time in total did you spend on sitting in leisure time in the past week?” Participants will be asked to further indicate the sedentary time for each behavior (playing cards, playing chess, reading, writing, socializing with friends or family, doing hobbies, driving, riding, time on public transport and any other activities). When two or more activities are carried out at the same time, only the time for the main activity should be counted. For example, if you are watching TV and doing crafts, then count it as TV time or craft time, but not both.

##### Self-Efficacy

Self-efficacy will be obtained through the Self-Efficacy for Exercise Scale (SEE), which is suitable for older adults and has been tested the validity and reliability by a previous study (35). The nine-item scale focuses on the confidence to participate in regular exercise when facing different situations. The SEE score, ranging 0–90, is scored by adding the score on each item (0–10), with higher scores indicating higher confidence levels on regular exercise.

##### Self-Regulation

The 12-item Physical Activity Self-Regulation scale (PASR-12) which is concise and validated for older adults will be used to assess self-regulation (36). It comprises 12 items addressing the self-regulatory strategies, involving the following dimensions: self-monitoring, goal setting, eliciting social support, reinforcements, time management, and relapse prevention. Each item has a score ranging from 1 to 5 and the PASR-12 can have a score ranging from 12 to 60.

##### Cognitive Function

Consistent with prior China Health and Retirement Longitudinal Study publications (37, 38), we will use the Telephone Interview for Cognitive Status (TICS-10), a questionnaire that assesses the individual's orientation, attention, episode memory (39, 40). Participants will be asked to reply the date (year, month, day), the day of the week, and season, serial subtractions of 7 from 100 five times, and immediate and delayed recall a list of Chinese nouns as many as they can. The orientation will be assessed by replying the date, the day of the week, and season, while the attention will be assessed by computing serial subtractions of 7 from 100 five times, and nouns recalling will be used to measure the episode memory. TICS-10 scores will be calculated by summing the three measures above, and the possible total scores range from 0 to 5 for orientation, 0 to 5 for attention, and 0 to 10 for episode memory with higher scores indicating better cognitive function.

##### Night-Time Sleep Quality

The night-time sleep quality dimensions (subjective sleep quality, sleep latency, sleep duration, habitual sleep efficiency, sleep disturbances, use of sleeping drugs, and daytime dysfunction) will be measured by the Pittsburgh Sleep Quality Index (PSQI) (41). The PSQI has been translated and adapted to Chinese populations, and has shown the validity and reliability in a previous study (42). Each response ranges from 0 to 3. Overall sleep quality scores will be calculated as the sum of these factors (0–21), with a higher score indicating poorer sleep quality.

*The following outcomes will be based on the anthropometry of each participant measured by trained technicians*.

##### Body Mass Index

Body mass index (BMI) is an individual's weight in kilograms divided by the square of height in meters. Participants will be required to be barefoot and wear light clothing when they are measuring the height and weight. Weight will be determined using an electronic scale, while height will be obtained by a portable stadiometer. Height and weight will be recorded to the nearest 0.1 cm and 0.1 kg, respectively.

##### Percentage Body Fat and Visceral Fat

Percentage body fat and visceral fat will be measured using Tanita BC-601 analyzer scales, with the participants wearing no shoes and socks.

##### Waist–Hip Ratio

We will obtain the waist–hip ratio through waist circumference divided by hip circumference. Hip circumference will be measured at the largest extension of the hips, while the waist circumference will be measured at the midpoint of the lowest rib margin and the upper margin of the iliac crest (43). Both the waist circumference and hip circumference will be recorded to the nearest 0.1 cm.

##### Systolic and Diastolic Blood Pressure

We will measure the blood pressure (BP) from the right hands of participants seated down by using a calibrated Omron U30 electronic sphygmomanometers. Strictly following the America Heart Association's Standardized protocol (44), three measurements should be taken with an interval of minimum 5 min, and the three readings will be averaged to be recorded as the systolic or diastolic BP.

#### Covariates

Socio-demographic characteristics, including sex (male/female), age, education level (illiterate, elementary, middle school, high school or above), marital status (married, never married, widowed, divorced), household income (<12,000, 12,000–19,999, 20,000–59,999, over 60,000), and employment (yes/no) will be collected at baseline.

Health behaviors including smoking status (never, former, current), alcohol consumption (never/seldom, <once a month, >once a month, daytime napping (0, 1–60 min/day, over 60 min/day) will also be obtained at baseline.

#### Statistical Analysis

Mean and standard deviation, frequency and percentage will be used to descript the continuous variables and categorical variables, respectively. Student *t*-tests and Chi-squared tests will be conducted to examine differences between two groups for continuous variables and categorical variables, respectively (Table 1). The difference-in-differences (DID) analysis will be performed to investigate the effect of the PA intervention by comparing the differences in outcomes over time between the intervention group and the control group (Table 2). The model will adjust for the baseline PASE score and covariates, which might differ between the two groups.

**Table 1:** Characteristics of eligible participants.

**Characteristics**	**Overall (*n*)**	**Intervention group (*n*)**	**Control group (*n*)**	***P*-Value**
Age, mean ± SD	–	–	–	–^a^
Male, *n* (%)	–	–	–	–^b^
BMI, mean±SD	–	–	–	–^a^
Employment, *n* (%)	–	–	–	–^b^
Yes	–	–	–	
No	–	–	–	
Household income (RMB), *n* (%)	–	–	–	–^b^
<12,000	–	–	–	
120,000–190,000	–	–	–	
20,000–59,999	–	–	–	
Over 60,000	–	–	–	
Education level, *n* (%)	–	–	–	–^b^
Illiterate	–	–	–	
Elementary	–	–	–	
Middle school	–	–	–	
High school or above	–	–	–	
Marital status, *n* (%)	–	–	–	–^b^
Married	–	–	–	
Never married	–	–	–	
Widowed	–	–	–	
Divorced	–	–	–	
Smoking status, *n* (%)	–	–	–	–^b^
Never	–	–	–	
Former	–	–	–	
Current	–	–	–	
Alcohol consumption, *n* (%)	–	–	–	–^b^
Never/seldom	–	–	–	
< once a month	–	–	–	
>once a month	–	–	–	
Daytime napping, *n* (%)	–	–	–	–^b^
0	–	–	–	
1–60 min/day	–	–	–	
Over 60 min/day	–	–	–	

*BMI, body mass index; SD, standard deviation*.

a *Student t-test*,

b *Chi-squared test, P values <0.05 are considered significant*.

**Table 2:** Coefficients of difference-in-difference regression model.

**Outcomes**	**Coefficient (95% CI)**	***P*-Value**
Primary outcome (mean)
PASE score	–	–
Secondary outcomes (mean)
Sedentary time	–	–
SEE average score	–	–
PASR-12 average score	–	–
TICS-10 average score	–	–
PSQI average score	–	–
BMI	–	–
Percentage body fat	–	–
Visceral fat	–	–
Waist–hip ratio	–	–
Systolic and diastolic BP	–	–

*PASE, Physical Activity Scale for the Elderly; SEE, Self-Efficacy for Exercise Scale; PASR-12, 12-item Physical Activity Self-Regulation scale; TICS-10, Telephone Interview for Cognitive Status; PSQI, Pittsburgh Sleep Quality Index; BMI, Body Mass Index; coefficients calculated from difference-in-differences model, adjusted for baseline PASE score and covariates, P values <0.05 are considered significant*.

## Discussion

PA not only plays a significant role in preventing a large number of age-related diseases, such as cognitive impairment, cardiovascular and metabolic diseases, but also reduces disability and improves the quality of later life in older adults (8, 45). For instance, PA was significantly associated with a 14–21% reduction in the risk of dementia among older adults (6). Nevertheless, most older adults are physically inactive (7), especially in rural China, where the PA level of rural older adults is significantly lower than their urban counterparts (46). The fact that the vast majority of Chinese rural older adults do not meet the PA recommendation (8) constitutes a daunting public health challenge. Therefore, conducting a PA intervention in rural China to improve PA is urgently needed for Chinese rural older adults.

The SAWA has several strengths. First, it is a multilevel PA intervention guided by the SEM, which proposed that multilevel interventions could be the most effective in changing behavior. Previous research showed that the multilevel approach (individual, interpersonal, and community levels) is a promising method for promoting exercise (28, 47, 48). Second, it will assess the maintenance by evaluating the longer-term effects at 6-, 12- and 24-month after the baseline. Third, SAWA will obtain self-efficacy and self-regulation for PA at every time point, which allows to determine the mechanism of the changes in PA. Fourth, the interventions were developed based on the findings of our previous survey in the same population, making the interventions more tailored. For instance, many rural older adults believed vigorous farming is beneficial in that survey. On the contrary, high intensity of PA at work (including farming) would increase the risk of cardiovascular events and mortality from all causes, known as the “physical activity paradox” (49). The misconception will thus be addressed in our training sessions. Fifth, we will provide participants with a variety of exercise options. Unlike other interventions which focus on promoting only one type of exercise (e.g. walking), participants in the study will be able to choose the type of exercise based on their preference and our recommendations. Sixth, SAWA not only targets the improvement of PA level, but also focuses on health status changes including changes in cognitive function, obesity, and night-time sleep quality.

The study also has several limitations. First, the use of the PASE scale as opposed to accelerometers to measure the PA level may introduce recall bias. Second, the seasonal variation of PA is not considered in the intervention design and data collection, which may pose a threat to the main findings. Third, the importance of the built environment in promoting regular PA in older adults has been demonstrated (50). However, such interventions, such as renovating and cleaning the pavement which participants walk the most, will not be implemented.

To our knowledge, little research on PA intervention among Chinese older adults has been conducted. The proposed study represents the first physical activity RCT intervention to be conducted of its kind. If the study produces positive results, it will be possible to recommend similar strategies to be implemented in other Chinese older adults and beyond.

## Ethics Statement

The study has been approved by the Sichuan University Medical Ethical Review Board prior to the start of the study (K2019073). The eligible participants will be required to sign the informed consent before intervention if they are willing to participate, and the participants will be informed that they can withdraw at any time.

## Author Contributions

JZ and NL: conceptualization. JZ: methodology, resources, validation, visualization, supervision, project administration, and funding acquisition. NL, YW, QD, and JH: investigation. NL: writing–original draft preparation. JZ, NL, YW, QD, and JH: writing–review and editing. All authors contributed to the article and approved the submitted version.

## Funding

This work was supported by the National Nature Science Foundation of China (Grant No. 71904135) and the China Postdoctoral Science Foundation (Grant No. 2020T130440).

## Conflict of Interest

The authors declare that the research was conducted in the absence of any commercial or financial relationships that could be construed as a potential conflict of interest.

## Publisher's Note

All claims expressed in this article are solely those of the authors and do not necessarily represent those of their affiliated organizations, or those of the publisher, the editors and the reviewers. Any product that may be evaluated in this article, or claim that may be made by its manufacturer, is not guaranteed or endorsed by the publisher.
